# Evaluation of Parental Knowledge and Practices in Pediatric Fever Management Among Iranian Families: A Cross-Sectional Study

**DOI:** 10.3390/pediatric18030061

**Published:** 2026-04-22

**Authors:** Tarlan Soumei, Sara Hamideh Kerdar, David D. Martin, Parviz Rafiezadeh, Ekkehart Jenetzky

**Affiliations:** 1 Institute of Integrative Medicine, Faculty of Health/School of Medicine, Witten/Herdecke University, 58455 Witten, Germany; tarlan_so@yahoo.com (T.S.); david.martin@uni-wh.de (D.D.M.); 2 Main-Vision Eye Center, 60329 Frankfurt, Germany; dr.rafizade@yahoo.com; 3 Federal Institute of Occupational Safety and Health, 44061 Dortmund, Germany; 4 Clinic for Paediatrics and Adolescent Medicine, University of Tübingen, 72076 Tübingen, Germany; 5 Department for Child and Adolescent Psychiatry and Psychotherapy, University Medical Center, Johannes Gutenberg University Mainz, 55099 Mainz, Germany

**Keywords:** health literacy, fever of unknown origin, antipyretics, acetaminophen, ibuprofen, pediatrics, Iranian people

## Abstract

**Background/Objectives**: Fever is a common concern among parents, often leading to heightened anxiety and misconceptions about its management. While fever phobia has been extensively studied in Western countries, data from the Middle East—particularly Iran—remain limited. Understanding parental knowledge and anxiety in this cultural context is essential for developing targeted educational interventions. This study aims to assess parental knowledge, behavior, and anxiety regarding fever in children and to identify factors associated with higher levels of anxiety among Iranian parents, thereby contributing culturally specific evidence to the international literature on pediatric fever management. **Methods**: A cross-sectional study was conducted involving 552 parents from Tehran, Iran, recruited through convenience sampling. Data were collected using self-administered questionnaires assessing demographic characteristics, knowledge about fever, treatment practices, and anxiety levels using a 10-point Likert scale. Principal component analysis (PCA) was performed to examine the underlying factors influencing parents’ decisions to reduce fever. Univariate and multivariate linear regression analyses on standardized z-values were conducted to determine the predictors of fever-related anxiety. **Results**: Results showed that 67.4% of parents experienced anxiety when managing their child’s fever, with 65.6% perceiving fever as harmful. Fear of febrile seizures (77.4%) and brain damage were significant concerns motivating parents to reduce fever. Female parental sex (β = 0.336, *p* = 0.004) and the perception of fever as harmful (β = 0.058, *p* < 0.001) were the strongest predictors of fever-related anxiety. The PCA identified two key factors influencing fever management behavior: well-being protection and medical risk prevention. Parents commonly treated fever using combinations with either Paracetamol or Ibuprofen (47.6%). **Conclusions**: Parental anxiety about fever in Iran is largely driven by misconceptions, especially regarding febrile seizures and brain damage. Culturally tailored education and clear communication from healthcare providers are essential to reduce these fears, improve fever management, and decrease unnecessary antipyretic use.

## 1. Introduction

Fever is a physiological response to infection, typically defined as a body temperature of ≥38 °C [1,2]. It is regulated by the hypothalamus and seldom exceeds 41 °C unless conditions like dehydration are present or the body’s heat-loss mechanisms are impaired [3]. Despite being a common and self-limiting symptom, particularly in viral infections among children [4,5], fever remains one of the primary reasons for pediatric medical consultations globally [5,6,7]. In some regions, such as Europe and the United States, up to 30% of pediatric visits are prompted by fever-related concerns, with similar trends observed in other countries [8,9].

Parents’ anxiety about fever often stems from misconceptions, with many fearing severe complications such as febrile convulsions or brain damage [10]. This phenomenon, termed “fever phobia” by Dr. Barton Schmitt in 1980, refers to an exaggerated fear of fever and a misunderstanding of its causes and consequences [11]. A recent systematic review confirmed that fever phobia remains a worldwide phenomenon, affecting caregivers across Asia, Europe, the Americas, Africa, and Australia, with low educational level, history of febrile seizures, and young maternal age as significant associated factors [12]. Parents often treat fever as a disease itself rather than as a symptom, leading to a propensity for premature and excessive antipyretic use, even at sub-febrile temperatures [13,14]. This tendency is driven by fears of complications such as brain damage, convulsions, dehydration, and even death, which have been consistently reported in studies from various countries, including the USA, UK, Canada, Saudi Arabia, Turkey, and Germany [13,15,16,17,18,19,20]. A recent qualitative meta-synthesis has further demonstrated that, despite decades of education and research, parental anxiety about fever persists globally, with parents continuing to perceive fever as a disease rather than a symptom [21]. Although recent research suggests that the primary reason for reducing fever should be to alleviate the child’s discomfort [22,23], parents often prioritize their own sense of safety, leading to unnecessary antipyretic use [24]. This frequent and sometimes excessive administration of antipyretics, especially at low temperatures, raises concerns, as fever can play a beneficial role in reducing bacterial and viral load during infections and potentially decreasing mortality in severe cases [23]. Importantly, antipyretics do not prevent febrile seizures, and high doses or frequent usage can result in toxicity or other adverse effects [23,25]. Mismanagement of antipyretics, including incorrect dosing, has been associated with an increased risk of overdose and harmful drug reactions [26,27].

Studies have shown that parental health literacy significantly influences their management of fever, as well as their decision-making regarding medical care [28,29]. Higher educational levels and access to accurate medical information can reduce fever-related anxiety and improve home management [28]. Conversely, inadequate knowledge often results in unnecessary medical consultations and aggressive interventions to lower body temperature [10,28,29]. Notably, a recent literature review from South-East and East Asia reported that 47–87% of parents exhibited moderate to high anxiety levels regarding childhood fever, with concerns about brain damage and intellectual disability being particularly prominent [30], suggesting that fever phobia transcends Western populations. In Iran, as in other countries, fever management is often accompanied by misunderstandings and anxiety [31]. Many parents believe that immediate temperature reduction can prevent severe outcomes like febrile seizures [32,33]. This highlights the need for tailored educational interventions to correct misconceptions and promote evidence-based fever management practices among caregivers.

Understanding the knowledge, attitudes, and practices of parents regarding pediatric fever is crucial to reducing unnecessary healthcare use and preventing improper treatment strategies. While this topic has been investigated in various Western and Middle Eastern countries, Iran-specific data remain scarce, despite cultural and healthcare system differences that may substantially influence parental fever management. This cross-sectional study aims to evaluate the level of parental knowledge, practices, and anxiety in managing pediatric fever in Tehran, Iran, providing culturally specific evidence to inform future educational and public health interventions.

## 2. Materials and Methods

### 2.1. Study Design and Participants

This cross-sectional study was conducted between February and May 2022 to assess parental knowledge and practices in pediatric fever management in Tehran, Iran. Data collection was carried out using two formats: paper-based and online questionnaires (Supplementary File S1). This study is reported in accordance with the Strengthening the Reporting of Observational Studies in Epidemiology (STROBE) guidelines for cross-sectional studies [34] (Supplementary File S2). The recruitment strategies and data collection methods differed for each format and are described separately below.

The study protocol, including the questionnaire and consent form, received ethical approval from the Ethics Committee of Witten/Herdecke University (S-145/2021). Participation was voluntary, and written informed consent was obtained from all participants before their inclusion in the study. Data were pseudonymized to protect participant confidentiality, and no identifiable information was stored or shared with third parties.

### 2.2. Paper Format

A convenience sampling method was employed to recruit participants for the paper-based questionnaire. The study team visited four public parks, two hospitals, one children’s entertainment playground, and one game center, all located in Tehran. Parents who were present at these locations during the study period were approached by the researchers and invited to participate in the survey. Those who agreed were provided with detailed written information regarding the study’s objectives, methods, and ethical considerations, followed by a consent form. Upon signing the consent form, participants were given the questionnaire to complete. The inclusion criteria for participation were the ability to read and write in Farsi and being a parent of at least one child younger than 14 years old. No incentives were offered for participation. In total, 437 parents completed the paper-based questionnaire at the above-mentioned locations. The total number of parents who were approached but declined participation was not systematically recorded.

### 2.3. Online Format

In parallel, an online version of the questionnaire was distributed via schools, kindergartens, and children’s art institutes. A list of potential institutions was identified through an internet search, and 27 institutions were contacted via telephone or WhatsApp. The study and its objectives were explained to administrators, and four institutions (two schools, one kindergarten, and one art institute) agreed to cooperate. These institutions distributed the questionnaire link through parental WhatsApp groups. A total of 115 parents completed the online questionnaire. Identical to the paper format, the online survey began with an explanation of the study, followed by a consent form before the questionnaire. Inclusion criteria for the online participants were identical to those for the paper-based participants. No incentives were provided for online participation either.

### 2.4. Questionnaire Development

The questionnaire was developed by reviewing relevant literature and adapting an existing questionnaire from a study conducted on German parents by Hamideh Kerdar et al. [24]. The original German questionnaire was translated into Farsi using a back-translation process to ensure linguistic accuracy and cultural appropriateness. A researcher fluent in both languages first translated the questionnaire from German to Farsi. The Farsi version was then translated back to German by a third-party fluent in both languages. The original authors of the German study reviewed the back-translated version and provided feedback, leading to minor adjustments in the Farsi version. Following the original German study [24], the questionnaire items were structured along the dimensions of knowledge, behavior, and experience, consistent with the Knowledge, Attitudes, and Practices (KAP) framework commonly used in health behavior research [35].

A pilot test was conducted with 12 parents to assess the comprehensibility of the questionnaire and identify any cultural adaptations needed. Parents who met the study’s inclusion criteria were asked to provide feedback on the clarity of the questions. Based on this feedback, further cultural adjustments were made, including the addition of a question regarding footbaths, which emerged as a common fever management practice among Iranian parents. Data from the pilot study were not included in the final analysis.

The final version of the questionnaire comprised 46 questions, divided into six demographic questions, 15 multiple-choice questions, nine triple-choice questions, and five scale or numerical questions related to body temperature. Additionally, 11 questions included an option for free-text responses, allowing parents to provide further details if desired. The medication-related questions focused on the use of paracetamol (acetaminophen), ibuprofen, or alternating between the two, asking parents to specify dosages, intervals of administration within a 24 h period, and whether they knew the correct dosage. Responses were recorded using fixed-choice options as well as a “don’t know” response. Location of temperature measurement was also assessed, and participants were allowed to select more than one option.

### 2.5. Data Collection and Analysis

Responses from both the paper-based and online questionnaires were manually entered into Microsoft Excel (2013) and then imported into SPSS version 22.0 (SPSS, Chicago, IL, USA) for analysis. In cases where responses were incomplete or missing, available cases were analyzed. Descriptive statistics were used to summarize participant demographics and key variables related to fever management practices. Categorical variables were presented as frequencies and percentages, while continuous variables were described using means and standard deviations.

For questions regarding antipyretic use, responses were categorized dichotomously. Answers indicating frequent or occasional use were coded as “yes,” while responses indicating rare or no use were coded as “no.” Regression analyses were performed to explore associations between parental knowledge and behavior in managing fever and demographic variables such as education level, number of children, and age of the oldest child.

### 2.6. Statistical Analyses

We followed a previously reported approach [24] to conceptualize anxiety as a continuum, ranging from confidence to anxiety. To capture this, parents rated their confidence or anxiety on a 10-point Likert scale, where 1 represents complete calm and security, and 10 indicates feelings of restlessness and anxiety when their child has a fever. This approach allowed us to understand a wider range of emotional responses. To standardize these anxiety scores, we applied a Z-transformation to the Likert scale data, normalizing the distribution for future analysis [36]. These standardized scores were subsequently used in regression models. For the purpose of categorical analysis, the scale was dichotomized into two groups: “confident” (scores 1–5) and “anxious” (scores 6–10). The perception of fever as either useful or harmful was assessed using a 10-point Likert scale, with 1 representing the belief that fever is beneficial and 10 indicating it is harmful. For regression analysis, the full scale was utilized, while for categorical comparisons, the data were divided into two groups: scores 1–5 (fever useful) and scores 6–10 (fever harmful).

Descriptive analysis was conducted on demographic variables and most questions to better understand the data distribution. An exploratory factor analysis with Varimax rotation was applied to identify patterns in responses to 11 reasons for reducing fever, with answers reported in binary format. Further correlation and univariate linear regression were performed to explore the relationships between key factors such as ‘anxiety levels,’ ‘knowledge,’ ‘behavior,’ and ‘experience.’ A multiple linear regression was then used to assess the connection between anxiety levels and significant variables identified in the univariate analysis. The level of significance was set at *p* < 0.05, and in cases of missing data, analyses were conducted using the available information. Data analysis was performed using IBM SPSS versions 25 and 26 (IBM, Armonk, NY, USA).

## 3. Results

### 3.1. Participant’s Characteristics

A total of 552 participants were included in the final analysis (Table 1), with the majority of responses collected via the paper-based questionnaire (79.2%). The sex distribution revealed a predominance of female participants, comprising 82.4% (*n* = 455), while 16.7% (*n* = 92) were male, and 0.9% (*n* = 5) did not disclose their sex. The mean age of mothers was 37.5 years (SD = 6.0), with a median of 38 years. Fathers had a mean age of 39.8 years (SD = 5.9), with a median of 39 years. Overall, the mean age of parents was 37.9 years (SD = 6.2), with a median of 38 years. The mean age of children was 2.1 years (SD = 0.9), with a median age of 2 years. Participants ranged in age from under 20 to over 50 years, with the largest age group being 30–39 years (51.4%, *n* = 284), followed by those aged 40–49 years (35.9%, *n* = 198). A small percentage of participants were younger than 20 years (1.3%, *n* = 7), while 5.6% (*n* = 31) were between 20 and 29 years, and 2.0% (*n* = 11) were 50 years or older. A portion of the respondents (3.8%, *n* = 21) did not report their age.

Regarding educational background, the majority of participants held a university degree, accounting for 84.6% (*n* = 467). A smaller proportion, 12.1% (*n* = 67), had completed college, while 1.4% (*n* = 8) had achieved a high school diploma or equivalent. Only 0.9% (*n* = 5) of participants had no formal education. Educational data were missing for 0.9% (*n* = 5) of participants.

Occupational status varied, with 38.0% (*n* = 210) working full-time, 21.0% (*n* = 116) working part-time, and 36.4% (*n* = 201) identifying as housewives. Unemployment was reported by 2.7% (*n* = 15) of the sample, and 0.7% (*n* = 4) were retired. Data regarding employment status were not provided by 1.1% (*n* = 6) of respondents.

In terms of family structure, the majority of participants had one child (63.2%, *n* = 349), while 28.6% (*n* = 158) reported having two children, and a small percentage had three (2.9%, *n* = 16) or four children (0.4%, *n* = 2). Information on the number of children was missing for 4.9% (*n* = 27) of respondents.

The age distribution of children showed that the largest group was aged 4–6 years, comprising 33.3% (*n* = 184) of the total. This was followed by children aged 7–9 years, representing 28.6% (*n* = 158), and those aged 1–3 years at 14.9% (*n* = 82). A smaller proportion of children were under 1 year old (1.6%, *n* = 9), while 13.6% (*n* = 75) were aged 10–14 years. A total of 44 cases (8.0%) had missing values (Table 2).

### 3.2. Fever Confidence/Anxiety

**Figure 1:**
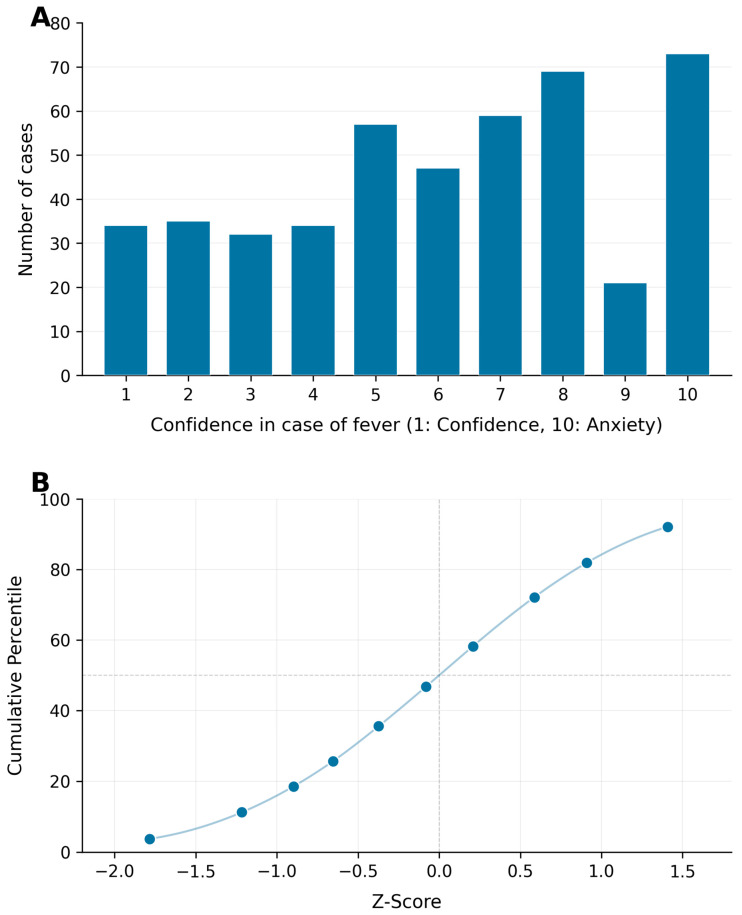
Parents’ responses to their child’s fever measured on two scales. (**A**) A 10-point Likert scale illustrating parental confidence (1) to anxiety (10) regarding fever management. (**B**) Z-scores reflecting the standardized distribution of confidence (negative values) and anxiety (positive values) in response to their child’s fever.

When examining the spectrum from complete confidence (score = 1) to anxiety (score = 10), the median level reported was 6 (IQR = 4), with a mean score of 5.74 (SD = 2.07). Figure 1A illustrates the distribution of responses on the 10-point Likert scale, while Figure 1B presents the corresponding standardized z-scores and percentiles. Lower z-scores or percentiles reflect higher confidence in managing fever, while higher z-scores indicate increased anxiety. In the binary analysis, 32.61% of participants expressed confidence when their child had a fever, while 67.39% reported feeling anxious.

### 3.3. Fever Management: Knowledge and Behavior

In terms of temperature assessment, 69.0% of parents primarily relied on thermometers to evaluate their child’s fever (Table 3). Additionally, 10.1% used a combination of hand-feeling and thermometer measurement, while 9.2% depended solely on hand-feeling. Few parents consulted a physician for temperature evaluation, with only 1.6% using this method. The remaining parents combined various approaches, including the use of both thermometer and doctor consultation (1.1%), or a combination of feeling, thermometer, and physician assessment (1.6%).

Regarding the location of temperature measurement, most parents reported using the axillary (armpit) region (44.7%) and the forehead (44.7%) as the primary sites for assessing body temperature. Oral measurements were used by 18.7% of parents, while 12.3% opted for the ear. Rectal temperature measurement was the least utilized method, with only 1.6% of parents using this method.

**Figure 2:**
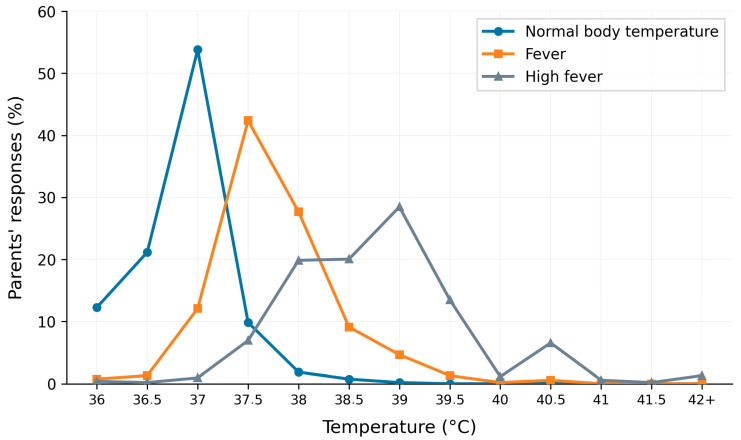
Schematic diagram illustrating parents’ awareness and understanding of normal and elevated body temperature in children.

**Figure 3:**
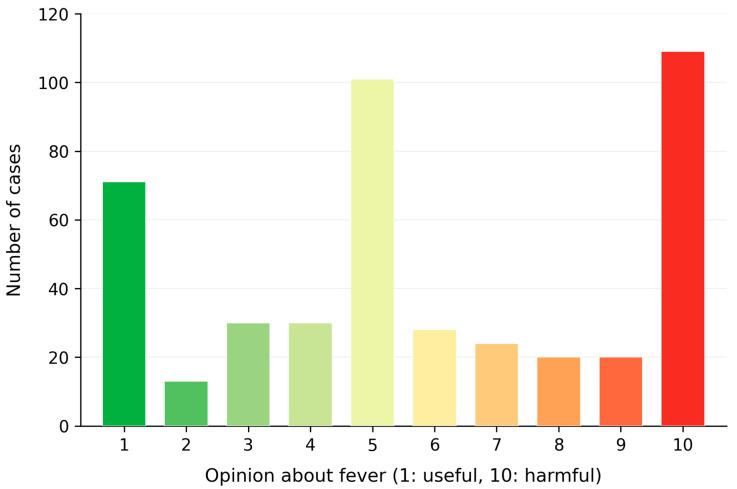
Parents’ knowledge of fever, represented on a 10-point Likert scale, where 1 indicates that fever is viewed as useful and 10 indicates that fever is considered harmful. The histogram shows the distribution of responses among participants.

When asked about their understanding of fever, 52.5% of parents correctly identified 37 °C as the normal body temperature, while 41.3% associated 37.5 °C with the onset of fever (Figure 2). Additionally, 39.5% believed that untreated fever could escalate to 40 °C. Despite their understanding, 77.4% of parents expressed fear of seizures as a primary risk of untreated fever, followed by concerns about dehydration (12.3%), serious illness (37.9%), and brain damage (44.7%). On the topic of whether fever is considered useful or harmful, the average score was 5.34 (SD = 1.79) (Figure 3). Overall, 34.42% of parents viewed fever as beneficial, while 65.58% regarded it as harmful.

On the subject of taking measures to treat a child’s fever, a substantial proportion (40.8%, *n* = 225) reported not using any method (Table 4). Among those who treated fever, Paracetamol was the most frequently used as a standalone treatment, with 5.4% (*n* = 30) of parents reporting its use. Ibuprofen was used as a sole treatment by 0.4% (*n* = 2) of parents, and natural/homeopathic remedies (such as herbal teas or homeopathic medications) were used by 0.2% (*n* = 1). Other methods, including techniques such as cold compresses or footbaths, were reported by 0.4% (*n* = 2) of parents. The most common approach to fever management involved combinations that included either Paracetamol or Ibuprofen. This approach was used by 47.6% (*n* = 263) of parents, indicating a preference for combining fever-reducing medications with other treatment methods, such as natural remedies or other supportive techniques. Combinations without Paracetamol or Ibuprofen, using only alternative treatments (e.g., antibiotics, natural remedies, or compresses), were rare, reported by only 0.7% (*n* = 4) of parents. A small group of parents (0.5%, *n* = 3) reported alternating between Paracetamol and Ibuprofen, a common strategy for managing fever in children. Finally, 4.0% (*n* = 22) of parents either did not know or did not provide a response regarding their fever treatment practices.

Most parents (75.9%, *n* = 419) indicated that their understanding of fever management was shaped by pediatricians. Family members also played a significant role in shaping these beliefs (40.6%, *n* = 224). Other sources included the internet (29.7%, *n* = 164), books (15%, *n* = 83), and advice from schools and kindergartens (7.8%, *n* = 43). A smaller number of parents (4.3%, *n* = 24) gained knowledge from alternative sources such as television, personal experiences, or guidance from a doctor’s office staff. About 3% (*n* = 17) of participants mentioned university education as a source of their fever-related knowledge.

### 3.4. Reasons for Reducing Fever

Parents reported a variety of reasons for reducing their child’s fever, often selecting multiple motivations. The most common concern was the prevention of febrile seizures (77.4%, *n* = 427). Additionally, 44.7% (*n* = 247) aimed to prevent potential brain damage. A similar proportion of parents, 37.7% (*n* = 208), sought to prevent other injuries caused by an excessive rise in body temperature. Other reasons included improving their child’s physical condition (30.3%, *n* = 167) and mental state (18.3%, *n* = 101), as well as encouraging better fluid intake (12.3%, *n* = 68). Nearly a third of parents (36.8%, *n* = 203) reduced fever to alleviate restlessness, while 9.1% (*n* = 50) indicated that fever reduction helped their child participate better in daily life. Some parents, 17.8% (*n* = 98), reported reducing fever to increase their own sense of security, and a smaller group (7.1%, *n* = 39) did so to promote faster recovery from the underlying illness for their child or other reasons (1.3%, *n* = 7).

To better understand the underlying motivations behind parents’ reasons for reducing fever, we conducted a principal component analysis (PCA) with Varimax rotation on the 11 response options. Two distinct factors were identified, using the criteria of an Eigenvalue of at least 1 and the scree plot of response options, which together accounted for 46.8% of the variance. Table 5 presents the frequency of responses and the loadings of each variable on the two components. In this analysis, Factor 1 primarily captures aspects related to well-being protection, with the highest loadings on reasons such as improving physical and psychological well-being, reducing strain on the child, and enhancing participation in daily life. This factor explains a substantial portion of the variance related to actions parents take to improve their child’s overall comfort and quality of life during a fever episode. Factor 2, on the other hand, focuses on medical risk prevention, with variables such as preventing febrile seizures, brain damage, and long-term damage due to high temperatures loading highly on this factor. This component represents parental concerns related to mitigating serious medical risks associated with fever. Interestingly, the item “Improve physical well-being” loaded moderately on both factors, indicating that parents perceive the improvement of physical well-being as related to both overall comfort and the prevention of potential medical risks. Similarly, some overlap is observed in other variables, such as the “reduction of strain on the child,” which also correlates with both factors but is more closely associated with well-being protection.

### 3.5. Understanding Elements of Fever Confidence/Anxiety

To explore the relationship between fever confidence/anxiety and various factors such as knowledge, behavior, experience, and demographics, we first performed a univariate linear regression analysis. The dependent variable was the standardized level of anxiety (z-value), and the analysis examined the impact of different elements on fever management and parental anxiety.

In the univariate analysis, two variables were found to be significant: parental sex (*p* = 0.013) and perception of fever as useful or harmful (*p* < 0.001). These two significant factors were then included in a multivariate linear regression analysis. The results of this analysis showed that parental sex (β = 0.336, 95% CI: 0.106 to 0.565, *p* = 0.004) and perception of fever as useful or harmful (β = 0.058, 95% CI: 0.031 to 0.085, *p* < 0.001) remained significant predictors of fever anxiety. Additionally, the perception of fever being harmful is associated with greater levels of anxiety. No other factors were found to be significant in either univariate analyses (Table 6).

### 3.6. Sex Differences in Fever Knowledge, Awareness, and Practices

To explore potential sex differences, mothers (*n* = 455) and fathers (*n* = 92) were compared across all major study domains (Table 7). Mothers reported significantly higher mean anxiety scores than fathers (6.21 ± 2.77 vs. 5.39 ± 2.61; *p* = 0.010), and a significantly greater proportion of mothers were classified as anxious (60.2% vs. 45.2%; *p* = 0.015). Regarding fever treatment, mothers more frequently used combination treatments with Paracetamol or Ibuprofen (52.7% vs. 34.5%; *p* = 0.003), while fathers showed a non-significant trend toward opting for no treatment (51.7% vs. 40.5%; *p* = 0.069). Mothers more frequently cited pediatricians as their primary source of information (78.7% vs. 65.2%; *p* = 0.008). No statistically significant sex differences were observed in the perception of fever as harmful, reasons for reducing fever, temperature measurement sites or methods, or the fever threshold for medication use. A trend toward significance was observed for improving fluid intake as a reason for fever reduction, which was more frequently endorsed by mothers (14.5% vs. 6.0%; *p* = 0.052).

## 4. Discussion

Parental knowledge and behavior are crucial in managing pediatric fever, as parents are typically the first to respond when their child becomes ill. In this cross-sectional study, we aimed to assess parental knowledge, behavior, and the associated anxiety regarding fever in Iran, following similar approaches used in other countries [13,15,16,17,18,19,20,24]. Interestingly, while the majority of parents expressed understanding of fever’s purpose, there remains a significant portion of parents who regard it with anxiety and uncertainty.

The high level of fever-related anxiety among Iranian parents (67.4%) aligns with the long-standing concept of “fever phobia,” initially identified by Kramer et al., 1985 [15]. In their foundational study, 94% of American parents expressed similar fears about fever’s potential to cause harm [15]. However, the focus of Iranian parents on the risk of brain damage and febrile seizures (77.4%) as primary drivers of anxiety stands out as a cultural distinction. In contrast, studies in Canada and the United Kingdom reported a broader spectrum of concerns, including dehydration and convulsions, but with less emphasis on brain damage [16,18]. A notable difference in the Iranian context was the reliance on axillary (44.7%) and forehead measurements (44.7%), while rectal measurement was rarely used (1.6%). This contrasts sharply with Canadian and German studies, where rectal measurement was more common due to its higher accuracy [18,24]. The preference for non-invasive methods in Iran may reflect cultural attitudes toward modesty and discomfort, which influence parental decision-making. This unique behavior underscores the need for culturally tailored education about the benefits and limitations of different fever measurement techniques. The mean fever threshold for antipyretic use among Iranian parents (37.85 °C) indicates a low tolerance for fever, comparable to findings in New Zealand and Germany, where parents often initiated treatment at similarly low thresholds [13,24]. However, unlike German parents who cited discomfort as the primary reason for fever treatment, Iranian parents predominantly aimed to prevent febrile seizures and brain damage. This highlights a gap in understanding the physiological role of fever and the importance of symptom management over unfounded fears of complications. The frequent use of either Paracetamol or Ibuprofen (47.6%) among Iranian parents mirrors trends observed in Canadian and New Zealand studies. However, the widespread use of antipyretics at sub-febrile temperatures in Iran reflects a cultural emphasis on precautionary measures, potentially fueled by misinformation or inadequate healthcare guidance [19]. This contrasts with the findings of Karwowska et al., where Canadian parents demonstrated higher confidence in managing fever without immediate recourse to medication [18]. The fear of febrile seizures reported by Iranian parents (77.4%) echoes findings in Turkey, where cultural beliefs strongly linked fever with convulsions [20]. Similarly, Iranian parents’ reliance on traditional remedies, such as footbaths, while uncommon in Western countries, underscores the role of cultural practices in shaping fever management. This reflects a broader need to integrate culturally appropriate educational materials to address these unique concerns. Despite 69% of Iranian parents using thermometers, misconceptions about fever risks were prevalent. Similar patterns were noted in Crocetti et al.’s study, where U.S. parents displayed a paradoxical combination of technical knowledge and high anxiety [17]. However, Iranian parents reported higher reliance on pediatricians as their primary source of information (75.9%) compared to internet-based sources favored in New Zealand [13] and Germany [24].

One of the key findings of this study is the role of anxiety in fever management. Although fever is a normal physiological response to infection, parental anxiety can lead to unnecessary interventions [11,37]. Our results show that 67.4% of parents experience anxiety when their child has a fever, a finding that aligns with international studies showing that “fever phobia” is a common phenomenon worldwide [24]. It should be noted that the concept of “fever phobia,” while widely used in the literature since Schmitt (1980) [11], lacks a formal theoretical framework and is best understood as a descriptive term for the pattern of exaggerated parental concerns about fever [12]. Interestingly, parental sex was a significant predictor of fever-related anxiety, with mothers reporting higher levels of anxiety than fathers. The findings are in accordance with previous research, where mothers showed more concern regarding their children’s health [38,39]. The sex-stratified analysis (Table 7) provides further insight into these differences. Mothers reported significantly higher anxiety levels than fathers (mean 6.21 vs. 5.39; *p* = 0.010), with 60.2% of mothers classified as anxious compared to 45.2% of fathers (*p* = 0.015). In the Iranian cultural context, mothers typically assume the primary caregiving role, resulting in more direct exposure to their child’s illness episodes and greater responsibility for treatment decisions. This heightened sense of responsibility may amplify anxiety, as mothers may feel more accountable for health outcomes. The finding that mothers more frequently used combination treatments with Paracetamol or Ibuprofen (52.7% vs. 34.5%; *p* = 0.003) while fathers more often opted for no treatment at all (51.7% vs. 40.5%; *p* = 0.069) may reflect this dynamic: mothers’ higher anxiety may translate into a stronger urge to actively intervene. Similarly, mothers more frequently cited pediatricians as their primary source of information (78.7% vs. 65.2%; *p* = 0.008), suggesting greater engagement with professional medical guidance consistent with their primary caregiving role. Notably, no significant sex differences were found in the perception of fever as harmful, reasons for reducing fever, temperature measurement practices, or the fever threshold for medication. This suggests that while the underlying knowledge and beliefs about fever are comparable between mothers and fathers, the emotional response and resulting treatment behavior differ substantially, likely driven by gender caregiving norms.

Furthermore, the perception of fever as harmful was another significant factor associated with higher anxiety levels. The analysis showed that 65.6% of parents viewed fever as harmful, with concerns about potential long-term damage, brain damage, and febrile seizures. These findings align with other research, where parents commonly report febrile signs as changes in their child’s behavior and physical symptoms [5,7,33,40,41]. Fear of fever complications, such as seizures, brain damage, dehydration, and even death, strongly influences parents’ perception of fever as a threat to their child’s health [33,40,41]. Specifically, the fear of febrile seizures was one of the main reasons parents sought to reduce their child’s fever, as reported by 77.4% of participants. Rectal temperature measurement was the least utilized method, with only 1.6% of parents reporting its use. This could reflect general concerns about comfort and the potential invasiveness of the method [24,33,40,41]. The preference for less intrusive methods, such as axillary and forehead measurements, aligns with broader trends in healthcare, where parents prioritize ease and comfort when assessing their child’s health.

Another essential aspect of fever management is the method of treatment. While almost half of the parents in our study chose not to treat their child’s fever, 47.6% opted for combinations of Paracetamol or Ibuprofen, which raises concerns about potential misuse or overuse of antipyretics. Previous studies have suggested that antipyretic use is often driven by misconceptions about fever’s harms, which can lead to unnecessary treatment at lower fever thresholds [26,42]. In this study, parents reported using antipyretics at a mean fever threshold of 37.85 °C, which aligns with other findings in the literature that indicate parents may initiate treatment even at low-grade fever levels [24,43,44,45]. This raises the need for targeted educational campaigns that emphasize fever’s protective role and promote appropriate fever management strategies.

The principal component analysis (PCA) of parents’ motivations for reducing fever revealed, in contrast to three factors in Germany [24], two primary factors: well-being protection and medical risk prevention. These components reflect the dual motivations of parents, who aim to protect their child’s comfort and prevent potential medical complications. The significant overlap between the well-being protection factor and medical risk prevention suggests that parents often conflate concerns about comfort and safety, further complicating their decision-making process. This underscores the need for clear communication from healthcare providers, emphasizing that fever is generally beneficial and that reducing fever should prioritize the child’s comfort rather than mitigating unfounded fears. Parents often perceive the severity of their child’s condition in direct relation to the fever’s temperature [44,46], and this drives their need to control the situation to protect their children. Many parents feel a strong sense of responsibility in managing febrile episodes and actively seek to prevent situations that feel out of their control [5,7,40]. Parents’ relationships with healthcare professionals play a pivotal role in how they manage fever. Trust, empathy, and clear communication from healthcare providers are crucial in shaping parental attitudes toward fever management [40,46]. However, some parents also express concerns about the side effects and potential overdoses of antipyretics, although their fear of fever often outweighs these concerns [47,48]. The experience gained from previous febrile episodes contributes to a reduction in anxiety, as parents become more confident in recognizing signs of severity [5,33,47]. Many parents express the desire for more information regarding fever management and its implications [33,47,49], and they often look to healthcare providers as their primary source of information. External influences, such as the media and parental support groups, also play a role in shaping parental perceptions of fever [50]. Despite understanding fever’s role in fighting infections, the fear of adverse outcomes drives many parents to administer antipyretics or seek medical attention unnecessarily. Addressing these concerns through clear, empathetic communication and education could help reduce parental anxiety and promote more appropriate fever management strategies.

This study has several notable strengths. First, with a sample size of 552 parents, the study provides a substantial dataset for robust statistical analyses. Second, the use of both paper-based and online questionnaires enabled recruitment from diverse settings—including public parks, hospitals, playgrounds, and educational institutions—thereby capturing a broader cross-section of the urban population in Tehran. Third, the questionnaire was adapted from a validated instrument previously used in a German cohort [24], with rigorous back-translation and cultural adaptation, including a pilot test, which enhances cross-cultural comparability. Fourth, the application of principal component analysis to identify underlying motivational factors for fever reduction adds a novel analytical dimension rarely applied in comparable studies. Finally, this is one of the few studies to provide detailed data on parental fever management in an Iranian context, addressing a significant gap in the Middle Eastern literature.

Several limitations must be considered when interpreting the findings. First, convenience sampling was used, and the total number of parents approached but declining participation was not systematically recorded; therefore, a formal response rate could not be calculated, which limits the assessment of potential non-response bias. Second, the study was conducted in a single city, Tehran, which may limit generalizability to other regions of Iran, particularly rural areas, or to different cultural contexts. Third, the sample was predominantly female (82.4% mothers), which, while reflecting the predominance of mothers as primary caregivers, may limit the generalizability of findings to fathers and reduces the statistical power of sex-stratified analyses. Fourth, data were collected via self-report questionnaires, which are susceptible to social desirability and recall bias. Fifth, the cross-sectional design precludes causal inferences regarding the relationships between parental characteristics and fever-related anxiety. Finally, the situational context of data collection may have influenced responses; a parent accompanying a currently febrile child may respond differently from one without an acutely ill child. Future studies should consider prospective designs, broader geographic sampling, balanced sex-balanced recruitment, and assessment of actual fever management behavior in real time.

## 5. Conclusions

In summary, this study highlights the significant role of anxiety in parental fever management, with female sex and misconceptions about fever being key drivers of anxiety. Despite understanding fever’s utility, many parents remain anxious, leading to the overuse of antipyretics and unnecessary medical interventions. Educational interventions are crucial to addressing these misconceptions and improving parental confidence in managing fever effectively. Culturally tailored approaches should be considered, given the significant influence of family and cultural beliefs on parental practices. Future studies should further explore the relationship between parental anxiety, cultural beliefs, and fever management behaviors to develop effective educational strategies.

## Figures and Tables

**Table 1:** Participants’ demographic information.

Demographics	*N* (%)
Sex	
Female	455 (82.4)
Male	92 (16.7)
No answer/missing	5 (0.9)
Source of answers	
Paper questionnaire	437 (79.2)
Online questionnaire	115 (20.8)
Age (years)	
<20	7 (1.3)
20–29	31 (5.6)
30–39	284 (51.4)
40–49	198 (35.9)
≥50	11 (2.0)
No answer/missing	21 (3.8)
Education level	
No school certificate	5 (0.9)
High school	8 (1.4)
College	67 (12.1)
University degree	467 (84.6)
No answer/missing	5 (0.9)
Occupation	
Part-time	116 (21.0)
Full-time	210 (38.0)
Retired	4 (0.7)
Housewife	201 (36.4)
Without a job	15 (2.7)
No answer/missing	6 (1.1)
Number of children	
1	349 (63.2)
2	158 (28.6)
3	16 (2.9)
4	2 (0.4)
No answer/missing	27 (4.9)

**Table 2:** Age distribution of children.

Children’s Age (Year)	*N* (%)
<1	9 (1.6%)
1–3	82 (14.9%)
4–6	184 (33.3%)
7–9	158 (28.6%)
10–14	75 (13.6%)
No answer/missing	44 (8.0%)

**Table 3:** Methods of temperature assessment.

Temperature Assessment	*N* (%)
Contradictory	7 (1.3%)
Feel, e.g., hand	51 (9.2%)
Thermometer	381 (69.0%)
Physician	9 (1.6%)
Feel and thermometer	56 (10.1%)
Feel and thermometer and doctor	9 (1.6%)
Feel and doctor	5 (0.9%)
Thermometer and doctor	6 (1.1%)
No answer/missing	28 (5.1%)

**Table 4:** Fever treatment methods used by parents.

Fever Treatment Method	*N* (%)
No treatment	225 (40.8%)
Paracetamol only	30 (5.4%)
Ibuprofen only	2 (0.4%)
Natural/Homeopathic only	1 (0.2%)
Other methods only (e.g., compresses)	2 (0.4%)
Combination with Ibuprofen or Paracetamol	263 (47.6%)
Combination without Ibuprofen or Paracetamol	4 (0.7%)
Paracetamol/Ibuprofen alternating	3 (0.5%)
No answer/missing	22 (4.0%)

**Table 5:** Principal component analysis of reasons for reducing fever, showing loadings on two components: Factor 1 (Well-being Protection) and Factor 2 (Medical Risk Prevention).

Reasons for Reducing Fever	*N* (%)	Factor 1: Well-Being Protection	Factor 2: Medical Risk Prevention
Prevent febrile seizures	427 (77.4)	0.00	**0.53**
Prevent brain damage	247 (44.7)	0.07	**0.75**
Prevent long-term damage from excessive temperatures	208 (37.7)	0.21	**0.67**
Improve physical well-being	167 (30.3)	0.50	**0.57**
Improve psychological well-being	101 (18.3)	**0.63**	0.41
Improvement in fluid intake	68 (12.3)	**0.60**	0.22
Reduce strain on the child	203 (36.8)	**0.59**	0.32
Better participation in daily life	50 (9.1)	**0.79**	0.07
Increase parents’ sense of security	98 (17.8)	**0.67**	0.18
Faster recovery from the underlying illness	39 (7.1)	**0.73**	−0.13
Other reasons	7 (1.3)	0.23	0.04

Factor loading above 0.5 was marked in bold.

**Table 6:** Univariate and multivariate linear regression analysis of factors influencing fever anxiety, showing the relationships between demographics, experience, knowledge, behavior, and sources of information with the standardized level of anxiety. Significant variables in the univariate analysis (sex and perception of fever as useful or harmful) were included in the multivariate analysis. Positive ß-values indicate increased anxiety, while negative values indicate more confidence.

	Level of Anxiety						
	Univariate Linear Regression Analysis				Multivariate Linear Regression Analysis		
Variables	* N * (%)/Mean (SD)	R^2^	ß	* p * Value	ß	95% CI	* p * Value
Demographics	-	-	-	-	-	-	-
Education level (%)	-	-	-	-	-	-	-
No school certificate	5 (0.9)	0.001	0.338	0.559	-	-	-
High school	8 (1.4)	0.009	0.214	0.320	-	-	-
College	67 (12.1)	0.000	−0.037	0.788	-	-	-
University degree	467 (84.6)	0.001	−0.076	0.551	-	-	-
No answer/missing	5 (0.9)	-	-	-	-	-	-
Age (years) (mean)	38.00 (5.97)	0.002	−0.007	0.332	-	-	-
Sex (male vs. female) (%)	92 (16.7)	0.012	0.297	0.013	0.336	(0.106 to 0.565)	0.004
Experience (mean)	-	-	-	-	-	-	-
Number of children	1.38 (0.56)	0.000	0.019	0.813	-	-	-
Parenting experience (years)	5.25 (2.81)	0.000	−0.004	0.784	-	-	-
Knowledge (mean)	-	-	-	-	-	-	-
Definition of fever in °C	37.77 (0.61)	0.001	−0.058	0.434	-	-	-
Site of measurement (%)	-	-	-	-	-	-	-
Rectal	9 (1.6)	0.000	0.134	0.690	-	-	-
Oral	103 (18.7)	0.000	−0.018	0.872	-	-	-
Underarm	247 (44.7)	0.000	−0.026	0.767	-	-	-
Ear	68 (12.3)	0.005	0.227	0.094	-	-	-
Forehead	247 (44.7)	0.000	−0.015	0.867	-	-	-
Reason for reducing fever (mean)					-	-	-
Factor 1: Well-being Protection	0.008 (1.01)	0.000	0.002	0.967	-	-	-
Factor 2: Medical Risk Prevention	0.013 (1.00)	0.005	0.072	0.105	-	-	-
Temperature threshold for medication in °C (mean)	37.85 (0.66)	0.002	−0.59	0.385	-	-	-
Source of information (%)					-	-	-
Paediatrician (doctor)	419 (75.9)	0.001	0.063	0.567	-	-	-
Family	224 (40.6)	0.005	0.143	0.110	-	-	-
Internet	164 (29.7)	0.003	0.123	0.199	-	-	-
Fever is useful or harmful (mean)	5.77 (3.17)	0.037	0.060	<0.001	0.058	(0.031 to 0.085)	<0.001
Behaviour (%)	-	-	-	-	-	-	-
No treatment	225 (40.8)	0.001	−0.070	0.436	-	-	-
Paracetamol only	30 (5.4)	0.000	−0.071	0.712	-	-	-
Ibuprofen only	2 (0.4)	0.000	0.335	0.637	-	-	-
Natural/Homeopathic only	1 (0.2)	0.001	−0.756	0.450	-	-	-
Other methods only (e.g., compresses)	2 (0.4)	0.001	−0.394	0.579	-	-	-
Combination with Ibuprofen or Paracetamol	263 (47.6)	0.003	0.112	0.208	-	-	-
Combination without Ibuprofen or Paracetamol	4 (0.7)	0.002	−0.487	0.333	-	-	-
Paracetamol/Ibuprofen alternating	3 (0.5)	0.000	−0.273	0.638	-	-	-
No answer/missing	22 (4.0)	-	-	-	-	-	-

**Table 7:** Comparison of fever-related knowledge, anxiety, practices, and information sources between mothers and fathers. Continuous variables are presented as mean (SD) and compared using the Mann–Whitney U test. Categorical variables are presented as *n*/*N* (%) and compared using the Chi-square test or Fisher’s exact test, where expected cell counts were <5. Statistically significant values (*p* < 0.05) are marked with an asterisk (*).

Variable	Mothers (*n* = 455)	Fathers (*n* = 92)	*p*-Value
*Fever-related anxiety*			
Anxiety score, mean (SD)	6.21 (2.77)	5.39 (2.61)	0.010 *
Proportion anxious (≥6), *n/N* (%)	256/425 (60.2%)	38/84 (45.2%)	0.015 *
*Perception of fever*			
Useful vs. harmful score, mean (SD)	5.83 (3.20)	5.42 (3.05)	0.221
Fever perceived as harmful (≥6), *n/N* (%)	189/409 (46.2%)	34/85 (40.0%)	0.354
*Fever treatment approach*			
No treatment, *n/N* (%)	179/442 (40.5%)	45/87 (51.7%)	0.069
Combination with Ibuprofen/Paracetamol, *n/N* (%)	233/442 (52.7%)	30/87 (34.5%)	0.003 *
*Reasons for reducing fever, n/N (%)*			
Prevent febrile seizures	359/435 (82.5%)	67/84 (79.8%)	0.653
Prevent brain damage	209/435 (48.0%)	38/84 (45.2%)	0.725
Prevent damage from high temperature	171/435 (39.3%)	37/84 (44.0%)	0.491
Improve physical well-being	139/435 (32.0%)	27/84 (32.1%)	1.000
Improve psychological well-being	83/435 (19.1%)	18/84 (21.4%)	0.729
Improve fluid intake	63/435 (14.5%)	5/84 (6.0%)	0.052
Reduce strain on child	175/435 (40.2%)	28/84 (33.3%)	0.288
Better participation in daily life	41/435 (9.4%)	9/84 (10.7%)	0.869
Increase parents’ sense of security	84/435 (19.3%)	14/84 (16.7%)	0.679
*Sources of information, n/N (%)*			
Pediatrician	358/455 (78.7%)	60/92 (65.2%)	0.008 *
Family	184/455 (40.4%)	39/92 (42.4%)	0.817
Internet	133/455 (29.2%)	30/92 (32.6%)	0.602
*Temperature measurement site, n/N (%)*			
Axillary	208/455 (45.7%)	38/92 (41.3%)	0.509
Forehead	205/455 (45.1%)	41/92 (44.6%)	1.000
Oral	82/455 (18.0%)	20/92 (21.7%)	0.491
Ear	55/455 (12.1%)	13/92 (14.1%)	0.713
Rectal	8/455 (1.8%)	1/92 (1.1%)	1.000
*Temperature assessment method, n/N (%)*			
Thermometer	369/455 (81.1%)	79/92 (85.9%)	0.350
Hand-feeling	101/455 (22.2%)	21/92 (22.8%)	1.000
*Temperature thresholds*			
Fever threshold for medication, °C, mean (SD)	37.86 (0.62)	37.84 (0.83)	0.919
Definition of fever, °C, mean (SD)	37.75 (0.59)	37.84 (0.68)	0.242

SD = standard deviation. * *p* < 0.05.

## Data Availability

The raw data supporting the conclusions of this article will be made available by the authors on request.

## Supplementary Materials

The following supporting information can be downloaded at: https://www.mdpi.com/article/10.3390/pediatric18030061/s1, Supplementary File S1: applied questionnaire in English; Supplementary File S2: filled STROBE checklist.
