# Supplementary material for: Evaluation of Parental Knowledge and Practices in Pediatric Fever Management Among Iranian Families: A Cross-Sectional Study
Source: Pediatr Rep. 2026 Apr 22;18(3):61. doi: 10.3390/pediatric18030061 (PMC13108139; doi:10.3390/pediatric18030061)
Supplement: Supplementary file 1 [file pediatrrep-18-00061-s001.zip › pediatrrep-4173404-supplementary S1.pdf]

Participant Code:

Questionnaire: Parents' Views on Children's Fever

To evaluate this questionnaire anonymously, please create a personal code:

1) Please enter the first two letters of your own first name:
2) Please enter the last two digits of your year of birth:
3) Please enter the first letter of your father's first name:
4) Please enter the first letter of your mother's first name:

1) Please state your age:

____

2) What is your highest educational qualification?

- ■ No formal education
- ■ Below high school diploma
- ■ High school diploma
- ■ Bachelor's degree
- ■ Master's degree
- ■ Doctorate (PhD)

3) What is your employment status?

- ■ Housewife
- ■ Full-time employed
- ■ Part-time employed
- ■ Currently unemployed
- ■ Retired

4) Your gender:

- ■ Female
- ■ Male

5) How many children do you have?

- ■ 1
- ■ 2
- ■ 3
- ■ 4 or more

6) What body temperature in °C do you consider as normal? (Please mark one number)

■ 36	■ 36.5	■ 37	■ 37.5	■ 38	■ 38.5	■ 39	■ 39.5	■ 40	■ 40.5	■ 41	■ 41.5	■ 42

7) From what body temperature in °C do you consider it a fever? (Please mark one number)

■ 36	■ 36.5	■ 37	■ 37.5	■ 38	■ 38.5	■ 39	■ 39.5	■ 40	■ 40.5	■ 41	■ 41.5	■ 42

8) From what body temperature in °C do you consider it a high fever? (Please mark one number)

■ 36	■ 36.5	■ 37	■ 37.5	■ 38	■ 38.5	■ 39	■ 39.5	■ 40	■ 40.5	■ 41	■ 41.5	■ 42

9) If untreated, to what body temperature in °C do you think the fever can rise?
_____ °C

10) Have you ever seen a seizure caused by fever?
■ Yes
■ No
■ Don’t know
■ No answer

From this section onwards, please answer the questions only for one of your children, preferably for your youngest child (0–9 years).

1) Please state the age of your child: _____ months _____ years
2) Gender of your child:
■ Boy
■ Girl
3) What is the current weight of your child? _____ kilograms
4) Is your child currently ill?
■ My child is healthy
■ Yes, with fever
■ Yes, without fever (because of medication)
■ Yes, without fever (without medication)
5) In the past year, how many times has your child had a fever-related illness? Number: _____
6) How many days does a fever episode in your child usually last? Number: _____
Please indicate what you usually do when your child has a fever and you think it is fever that requires action. (Multiple answers possible)
1) How do you usually measure your child’s body temperature? (Multiple answers possible)
■ I do not measure it
■ By hand
■ With a digital thermometer
■ The doctor measures it
■ Other: ____________
2) Where do you usually measure your child’s body temperature?
■ Rectum (bottom)
■ Mouth

- Forehead
- Ear
- Armpit

3. Does your child go to kindergarten or school when he/she has a fever?
■ Often
■ Sometimes
■ Rarely
■ Never

4. From what body temperature in °C do you reduce your child's fever? (Please mark one number)

36	36.5	37	37.5	38	38.5	39	39.5	40	40.5	41	41.5	42+

5. Do you reduce your child's fever with paracetamol (acetaminophen, e.g. Jalinous, Mofid Pharma, etc.)?
■ Yes
■ No
■ Don't know

If your answer is yes:
5a. Please state the amount of paracetamol you give your child: (mg / ml)

mg 40 / ml 1.25	mg 80 / ml 2.5	mg 120 / ml 3.75	mg 160 / ml 5	mg 240 / ml 7.5	mg 320 / ml 10	mg 420 / ml 12.5	mg 480 / ml 15
¼ tsp	½ tsp	¾ tsp	1 tsp	1.5 tsp	2 tsp	2.5 tsp	3 tsp

Other: __________

5b. At what time intervals do you give paracetamol to your child?
■ Every 2 to 4 hours
■ Every 4 to 6 hours
■ Every 6 to 8 hours
■ Don't know
■ Other: __________

6. Do you reduce your child's fever with ibuprofen (e.g. Advil, etc.)? (mg / ml)
■ Yes
■ No
■ Don't know

If your answer is yes:
6a. Please state the amount of ibuprofen you give your child: (mg)

mg 50	mg 75	mg 100	mg 150	mg 200	mg 250	mg 300	mg 350

Other: __________

6b. At what time intervals do you give ibuprofen to your child?
■ Every 2 to 4 hours
■ Every 4 to 6 hours
■ Every 6 to 8 hours
■ Don't know
■ Other: __________

7. Do you alternate between paracetamol (acetaminophen, e.g. Jalinous, Mofid Pharma, etc.) and ibuprofen (Advil, etc.) when treating your child's fever?
■ Yes
■ No
■ Don't know

8) Do you treat your child’s fever with antibiotics?

■ Yes
■ No
■ Don’t know

If your answer is "Yes":
8a) From what body temperature (°C) do you start lowering your child’s fever? (Please mark one number)

36	36.5	37	37.5	38	38.5	39	39.5	40	40.5	41	41.5	42+

9) Do you treat your child’s fever with natural or homeopathic remedies?

■ Yes, please name the remedies: __________
■ No
■ Don’t know

10) When your child has a fever, do you sponge/bathe him or her?

■ No
■ Yes, with lukewarm water
■ Yes, with cold water
■ Yes, with warm water

11) Do you treat your child’s fever with other methods?

(Please specify, e.g., using a wet cloth, washing the body with warm/cold/lukewarm water, etc.)
■ Yes, please describe the methods: __________
■ No
■ Don’t know

12) What are your reasons for lowering your child’s fever? (Multiple answers possible)

■ Faster recovery from illness
■ Prevention of febrile seizures
■ Prevention of brain damage
■ Prevention of other harm from excessively high body temperature
■ Improvement of physical condition
■ Improvement of emotional condition
■ Improvement in fluid intake
■ Reduction of restlessness
■ Better participation in everyday life
■ Increasing my own feeling of security
■ For deep sleep
■ Other reasons, please specify: __________

13) Do you feel that you are well advised by your doctor about fever?

■ Yes
■ No
■ Don’t know

14) Do you feel that you are well advised by your doctor about fever-reducing medications?

■ Yes
■ No
■ Don’t know

15) How do you feel when your child has a fever? (Please choose one number between 1 and 10)

1 = You feel calm and secure
10 = You feel restless and afraid

1	2	3	4	5	6	7	8	9	10

16) In your opinion, is fever useful or harmful? (Please select one number between 1 and 10)

1	2	3	4	5	6	7	8	9	10

1 = Useful 10 = Harmful

17) From which sources have you received information about fever management? (Multiple answers possible)
■ Pediatrician
■ Family
■ University
■ Kindergarten/Daycare
■ Books
■ Internet
■ Other trainings
■ Other: __________

When my child has a fever, I usually…

1. Place my child in bed to rest
■ Never
■ Rarely
■ Sometimes
■ Often
■ Always

2. I like to know how high the fever is
■ Never
■ Rarely
■ Sometimes
■ Often
■ Always

3. Measure the body temperature
■ Never
■ Rarely
■ Sometimes
■ Often
■ Always

4. Use non-drug methods to reduce fever
■ Never
■ Rarely
■ Sometimes
■ Often
■ Always

5. Sleep in the same room with my child
■ Never
■ Rarely
■ Sometimes
■ Often
■ Always

6. Consult a doctor
■ Never
■ Rarely
■ Sometimes
■ Often

■ Always

7. At night, I wake my child up from sleep in order to check if he/she still has a fever.
■ Never
■ Rarely
■ Sometimes
■ Often
■ Always

8. I make sure that my child drinks enough fluids.
■ Never
■ Rarely
■ Sometimes
■ Often
■ Always

Thank you very much for answering this questionnaire!
