# Supplementary material for: Evaluation of Parental Knowledge and Practices in Pediatric Fever Management Among Iranian Families: A Cross-Sectional Study
Source: Pediatr Rep. 2026 Apr 22;18(3):61. doi: 10.3390/pediatric18030061 (PMC13108139; doi:10.3390/pediatric18030061)
Supplement: Supplementary file 1 [file pediatrrep-18-00061-s001.zip › pediatrrep-4173404-supplementary S2 - STROBE_Checklist_Filled.pdf]

STROBE Statement—Checklist of items that should be included in reports of ***cross-sectional studies***

	Item No	Recommendation	Reported in
**Title and abstract**	1	*(a)* Indicate the study's design with a commonly used term in the title or the abstract	Title; Abstract (Methods)
		(b) Provide in the abstract an informative and balanced summary of what was done and what was found	Abstract
Introduction			
Background/rationale	2	Explain the scientific background and rationale for the investigation being reported	Section 1, paragraphs 1–4
Objectives	3	State specific objectives, including any prespecified hypotheses	Section 1, last paragraph
Methods			
Study design	4	Present key elements of study design early in the paper	Section 2.1, first sentence
Setting	5	Describe the setting, locations, and relevant dates, including periods of recruitment, exposure, follow-up, and data collection	Sections 2.1–2.3
Participants	6	*(a)* Give the eligibility criteria, and the sources and methods of selection of participants	Sections 2.2 and 2.3
Variables	7	Clearly define all outcomes, exposures, predictors, potential confounders, and effect modifiers. Give diagnostic criteria, if applicable	Sections 2.4 and 2.6
Data sources/ measurement	8*	For each variable of interest, give sources of data and details of methods of assessment (measurement). Describe comparability of assessment methods if there is more than one group	Sections 2.4 and 2.5
Bias	9	Describe any efforts to address potential sources of bias	Sections 2.2–2.4; Limitations
Study size	10	Explain how the study size was arrived at	Sections 2.2 and 2.3
Quantitative variables	11	Explain how quantitative variables were handled in the analyses. If applicable, describe which groupings were chosen and why	Section 2.6
Statistical methods	12	*(a)* Describe all statistical methods, including those used to control for confounding	Section 2.6
		(b) Describe any methods used to examine subgroups and interactions	Section 2.6 (PCA, gender analysis in 3.6)
		(c) Explain how missing data were addressed	Section 2.5, last sentence; Section 2.6
		(d) If applicable, describe analytical methods taking account of sampling strategy	Not applicable
		(e) Describe any sensitivity analyses	Not applicable
Results			
Participants	13*	*(a)* Report numbers of individuals at each stage of study—e.g. numbers potentially eligible, examined for eligibility, confirmed eligible, included in the study, completing follow-up, and analysed	Sections 2.2, 2.3 and 3.1
		(b) Give reasons for non-participation at each stage	Section 2.2; Limitations
		(c) Consider use of a flow diagram	Not included (convenience sampling; number of refusals not recorded)
Descriptive data	14*	*(a)* Give characteristics of study participants (e.g. demographic, clinical, social) and information on exposures and potential confounders	Section 3.1, Tables 1–2
		(b) Indicate number of participants with missing data for each variable of interest	Tables 1–7 (No answer/missing rows)
Outcome data	15*	Report numbers of outcome events or summary measures	Sections 3.2–3.6, Tables 3–7, Figures 1–3
Main results	16	*(a)* Give unadjusted estimates and, if applicable, confounder-adjusted estimates and their precision (e.g., 95% confidence interval). Make clear which confounders were adjusted for and why they were included	Section 3.5, Table 6 (univariate and multivariate regression with 95% CI)

		(b) Report category boundaries when continuous variables were categorized	Section 2.6 (anxiety: 1–5 confident vs. 6–10 anxious; fever perception: 1–5 useful vs. 6–10 harmful)
		(c) If relevant, consider translating estimates of relative risk into absolute risk for a meaningful time period	Not applicable (cross-sectional)
Other analyses	17	Report other analyses done—e.g. analyses of subgroups and interactions, and sensitivity analyses	Sections 3.4 (PCA, Table 5) and 3.6 (gender comparison, Table 7)
Discussion			
Key results	18	Summarise key results with reference to study objectives	Section 4, paragraphs 1–2; Section 5
Limitations	19	Discuss limitations of the study, taking into account sources of potential bias or imprecision. Discuss both direction and magnitude of any potential bias	Section 4, Limitations paragraph
Interpretation	20	Give a cautious overall interpretation of results considering objectives, limitations, multiplicity of analyses, results from similar studies, and other relevant evidence	Section 4, Discussion; Section 5
Generalisability	21	Discuss the generalisability (external validity) of the study results	Section 4, Limitations (single city, predominantly female sample, convenience sampling)
Other information			
Funding	22	Give the source of funding and the role of the funders for the present study and, if applicable, for the original study on which the present article is based	Funding statement (no external funding)

**Give information separately for exposed and unexposed groups.*

Note: An Explanation and Elaboration article discusses each checklist item and gives methodological background and published examples of transparent reporting. The STROBE checklist is best used in conjunction with this article (freely available on the Web sites of PLoS Medicine at http://www.plosmedicine.org/, Annals of Internal Medicine at http://www.annals.org/, and Epidemiology at http://www.epidem.com/). Information on the STROBE Initiative is available at www.strobe-statement.org.
